# Role of Antioxidants and Natural Products in Inflammation

**DOI:** 10.1155/2016/5276130

**Published:** 2016-10-10

**Authors:** Palanisamy Arulselvan, Masoumeh Tangestani Fard, Woan Sean Tan, Sivapragasam Gothai, Sharida Fakurazi, Mohd Esa Norhaizan, S. Suresh Kumar

**Affiliations:** ^1^Laboratory of Vaccines and Immunotherapeutics, Institute of Bioscience, Universiti Putra Malaysia, 43400 Serdang, Selangor, Malaysia; ^2^Department of Human Anatomy, Faculty of Medicine and Health Sciences, Universiti Putra Malaysia, 43400 Serdang, Selangor, Malaysia; ^3^Department of Nutrition and Dietetics, Faculty of Medicine and Health Sciences, Universiti Putra Malaysia, 43400 Serdang, Selangor, Malaysia; ^4^Department of Medical Microbiology and Parasitology, Faculty of Medicine and Health Sciences, Universiti Putra Malaysia, 43400 Serdang, Selangor, Malaysia

## Abstract

Inflammation is a comprehensive array of physiological response to a foreign organism, including human pathogens, dust particles, and viruses. Inflammations are mainly divided into acute and chronic inflammation depending on various inflammatory processes and cellular mechanisms. Recent investigations have clarified that inflammation is a major factor for the progression of various chronic diseases/disorders, including diabetes, cancer, cardiovascular diseases, eye disorders, arthritis, obesity, autoimmune diseases, and inflammatory bowel disease. Free radical productions from different biological and environmental sources are due to an imbalance of natural antioxidants which further leads to various inflammatory associated diseases. In this review article, we have outlined the inflammatory process and its cellular mechanisms involved in the progression of various chronic modern human diseases. In addition, we have discussed the role of free radicals-induced tissue damage, antioxidant defence, and molecular mechanisms in chronic inflammatory diseases/disorders. The systematic knowledge regarding the role of inflammation and its associated adverse effects can provide a clear understanding in the development of innovative therapeutic targets from natural sources that are intended for suppression of various chronic inflammations associated diseases.

## 1. Introduction

In the early nineteenth century, inflammation has become one of the most serious and interesting investigation research areas among biomedical researchers. The microcirculation is the main playground where the process of inflammatory cascade was evaluated and analyzed [1]. Inflammation includes a long chain of molecular reactions and cellular activity, which are designed to restore a tissue from simple skin cut or to repair tissue after giving birth or to cure several burn injuries. An inflammatory process in cellular and tissue levels includes a series of occasions with dilation of venules and arterioles, enhanced blood vessel permeability, and blood flow with percolation of leukocytes into the tissues [2]. Moreover, a dysfunction of tissue via proteolytic activity and regeneration of new humoral production for cell growth and reformation of novel functional and connective tissue are observed through a typical inflammatory response [1]. An inflammation cascade, which does not reach to resolution state, contributes to organ disorder and death. The inflammatory cascade is preprogrammed and stereotyped, and it is the only identified mechanism for restoration of tissue after injury. In the recent years, inflammation is one of the major target research areas among biomedical researchers, which includes various cellular processes (e.g., phagocytosis, chemotaxis, mitosis, and cell differentiation) [1]. There are several studies described on how the immune system (cellular immunity and antibodies) leads to an inflammatory response, and there is an additional huge clinical literature about individual steps in inflammation [1, 3].

## 2. Classification of Inflammation

**Figure 1:**
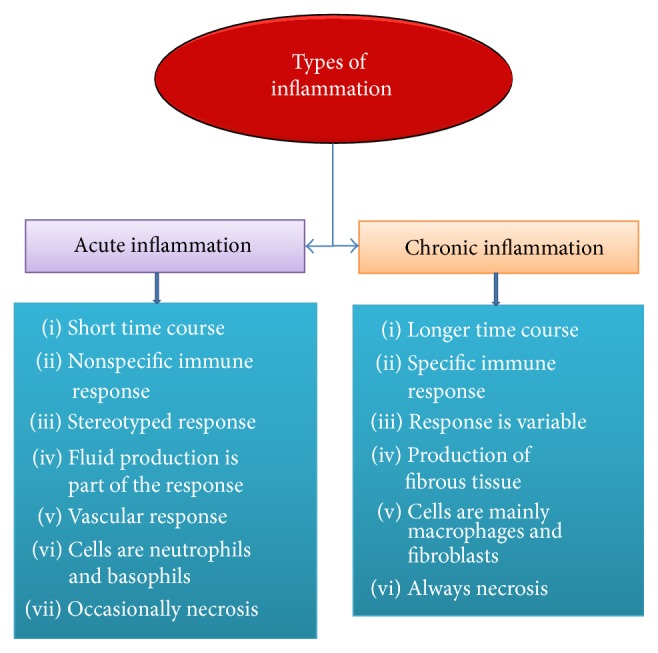
Classification of inflammation categorized by duration and immune functions.

Stages of inflammation depend on the duration of the process as well as various immune factors and inflammation has been classified into two different classes which are acute and chronic processes (Figure 1).

### 2.1. Acute Inflammation

**Figure 2:**
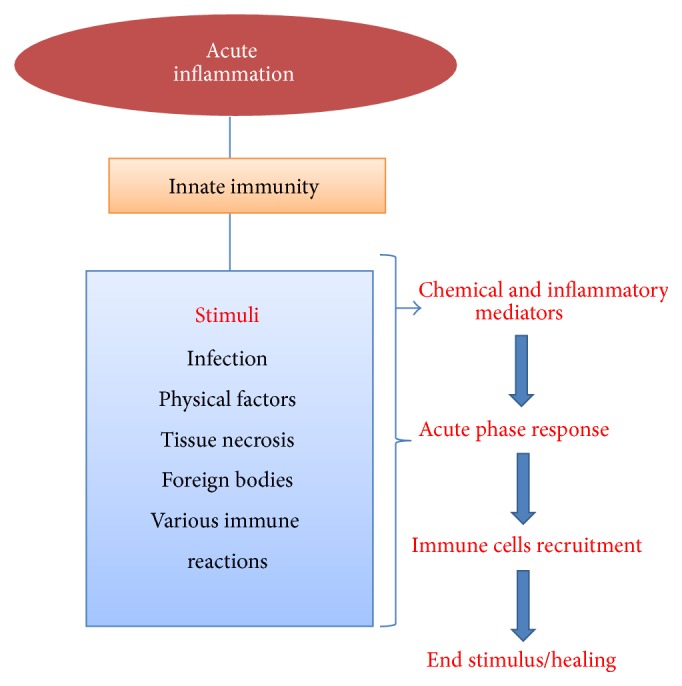
Acute inflammatory pathways and their activation process.

Acute inflammation is a short procedure, lasting from minutes to a few days, and its major features are leakage of plasma proteins or fluid and movement of leukocytes into an extravascular area [3]. These cellular and vascular reactions are intermediated by chemical factors produced from cells or plasma and are responsible for the classic clinical symptoms of inflammation such as swelling, redness, pain, warmth, and loss of function. Even though an inflammatory response can happen in any injurious stimulus, the characteristic of this process is the reaction of vascularized connective tissue [3]. There are three main steps in acute inflammatory responses which include enhanced blood flow to inflame area, followed by vasodilatation and enhanced vascular permeability with leakage of plasma from the microcirculation, and phagocytic leukocyte migration to the surrounding tissue [1, 3, 4] (Figure 2).

The first alterations noticed during inflammation are changes in the vascular flow and alterations in the caliber of small blood vessels. Newly generated capillary vessels and larger arterioles enhance the blood flow to this region. Progressive alterations in the endothelium boost the vascular permeability of the microvasculature, causing an escape of the fluid into an extravascular area which acquires the process to the next step [3]. The reduced volume of fluid in the lumen of blood vessels improves the viscosity of the blood and decreases the flow rate. Finally, after these changes in blood circulation, the margination of leukocytes begins and leukocytes adhere to the endothelium, at first, by rolling and then adhesion. Subsequently, they move through the vascular wall (transmigration) into the interstitial tissue. At this stage, the crucial purpose of acute inflammation, which is supplying leukocytes and plasma mediators to the injured area, is obtained [3, 4].

Based on the type of infection (bacteria, virus, parasite, etc.) the sensors, mediators, and target tissues are differed during inflammatory response [5]. For example, bacterial pathogens are recognized via receptors of the innate immune system like Toll-like receptors (TLRs) that are expressed in tissue-resident macrophages and stimulate the generation of inflammatory cytokines (e.g., TNF-*α*, IL-1*β*, and IL-6), chemokines (e.g., chemokine C-C motif ligand 2 and chemokine CXC motif ligand 8), and PGE2 [6]. These inflammatory productions then react on target tissues which include local blood vessels to stimulate vasodilation, extravasation of neutrophils, and leakage of plasma into the infected site (acute inflammation). Then, neutrophils tissue-resident macrophages and mast cells find the pathogens to eliminate them. This process is assisted by components of plasma and initiated by antibodies and its components. Classical pathway of complement is initiated by producing antibodies by natural antibodies, C-reactive protein, or serum amyloid protein [5]. Basically, microbes activate the classical pathway of complement through binding to C1q or mannose-binding lectin. Then, for the generation of the C3 convertase in classical pathway C2 and C4 components are required. C3 convertases cleave C3 to C3a and C3b and C3b then leads to the formation of the C5 convertase which generates C5a and C5b. Following that, C5b suppressed the production of C5b-9 terminal complement complex and then incorporated into bacterial cell walls and lysed them [3, 6].

These extremely powerful mediators and effectors do not discriminate among microbial and host targets; thus, harm to host tissues is inescapable [7, 8]. An effective acute inflammatory reaction results in the removal of infectious factors followed by a repair and resolution stage that is mediated via tissue-resident macrophages [9]. The shift in lipid markers from proinflammatory prostaglandins to lipoxins, which have anti-inflammatory effects, is vital for the changes from inflammation to resolution. In this stage, lipoxins decrease the role of neutrophils and, alternatively, activate the role of monocytes, which eliminate dead cells and trigger tissue remodeling. Protectins and resolvins which constitute another class of lipid marker in addition to TGF-*β* and other growth factors generated from macrophages also have a vital role in the resolution of inflammation including the foundation of tissue repair [9, 10].

### 2.2. Chronic Inflammation

**Figure 3:**
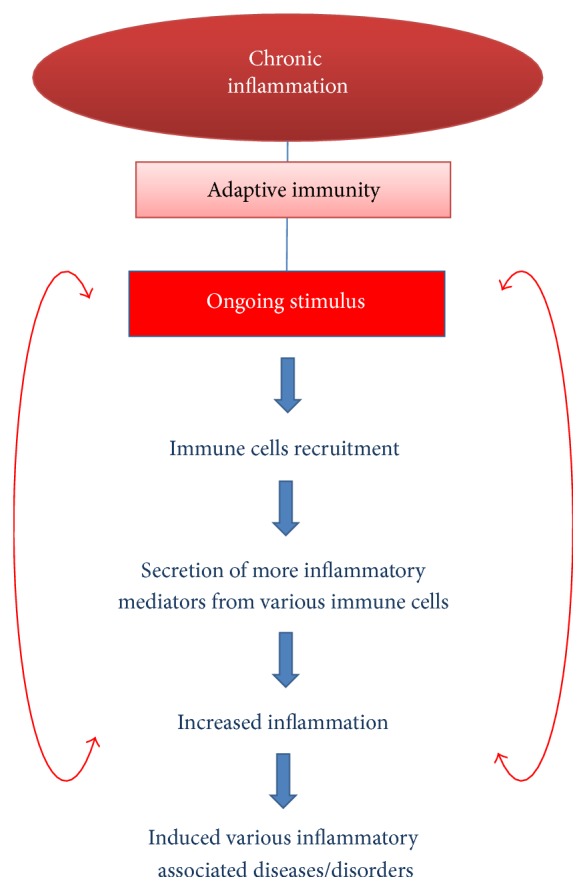
Steps involved in the chronic inflammatory processes and their consequences.

Inflammation is a vital response of human immune system. Nevertheless, the state of chronic inflammation can have several secondary consequences in biological response associated with enhanced risk of chronic diseases and disorders. Chronic inflammation in tissue usually happens when inflammatory responses are in the absence of an actual stimulus. It usually occurs through infections that are not resolved either within endogenous protection mechanisms or via some other resistance mechanism from host defences [11]. They can also happen from physical or chemical agents, which cannot be broken down, as well as from some kind of genetic susceptibility. Persistence of foreign bodies, continuous chemical exposures, recurrent acute inflammation, or specific pathogens are all crucial reasons for chronic inflammation [12] (Figure 3).

Molecular and cellular process of chronic inflammation is varied and depends on the type of inflamed cells and organ [13]. For example, in Western countries, chronic liver disease (CLD) mainly is related to chronic hepatitis B, hepatitis C infection, metabolic disease, alcohol consumption, drug/toxin-induced liver injury, and/or autoimmune causes, which include immune-mediated biliary disease. Hence, CLD is an important public health burden and is a main cause of worldwide morbidity and mortality [14]. Hepatic inflammation is one of the common types of liver illnesses and is known as the major driver of hepatic tissue damage contributing to fibrogenesis and hepatocellular carcinoma [15]. The inflammatory response throughout CLD can lead to innate immune system, which is the initial step of the body immune system toward invading pathogens and is vital for the overall survival of the host body. Liver innate immune cells include Kupffer cells, neutrophils, monocytes, dendritic cells, natural killer T cells, and natural killer cells and initiate and maintain hepatic inflammation through cytokine generation [16]. An unregulated cytokine production balance after liver injury can lead to hepatocytes cell death, which is a key discovery in several acute and chronic liver illnesses [17]. Inflammatory process which is too strong or fails to reach to resolution stage will become chronic and it will lead to the loss of the large amount of hepatocytes, leading to liver parenchyma damage [18]. Since myofibroblast can regenerate from hepatic stem cells, which can be replaced by dead hepatocytes, this can lead to an unresolved inflammatory response that can stimulate fibrotic/cirrhotic which can be distinguished through an irreversible decrease in liver function test [19]. A usual molecular feature of hepatitis is presence of damage of liver tissue and inflammatory cells.

## 3. Chronic Inflammation and Diseases

**Figure 4:**
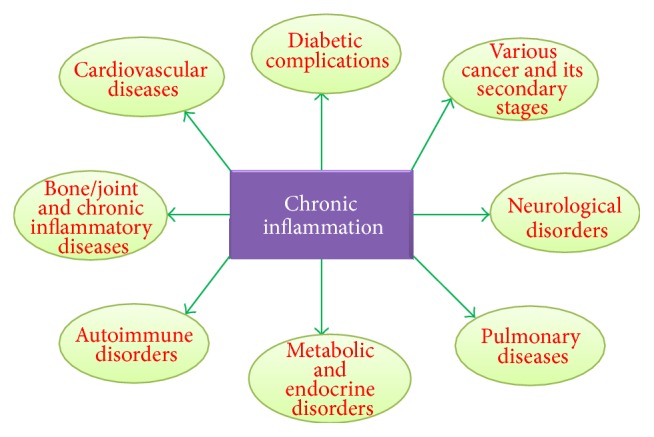
Chronic inflammation associated diseases/disorders due to longer term course of inflammation and various immune reactions.

Inflammation exists in patients with infections, environmental diseases (asbestos exposure and smoke inhalation, etc.), immune diseases, and chronic diseases like diabetes, gout, rheumatoid arthritis, cancer, and so forth. Nowadays, it has also been evident that a variety of illnesses have shown inflammatory response such as venous and chronic arterial diseases [20–22], myocardial ischemia [23, 24], Alzheimer's disease, acute cerebral stroke [22], cancer, and arterial hypertension [25]. Since many decades ago, there are also a diversity of clinical observations and molecular data for inflammation in osteoarthritis [26, 27]. In addition, there are some indicators of inflammation in highly depressed patients [28]. The numbers of illnesses, which are related to molecular mediators of inflammation, are large and expanding. Medical researchers have also proved that inflammation is related to obesity [29, 30] and insufficient typical physical exercise [31]. Currently, inflammation has become a vital topic for the study of human illness. Anti-inflammatory compounds, which have proven to be useful in one particular disease, could possibly turn out to be useful in another disease. Interestingly, it could open a huge range of possibilities for intervention in utilizing anti-inflammatory compounds [1] (Figure 4).

## 4. Role of Antioxidants in Inflammation

### 4.1. Free Radicals

A free radical is a molecule or atom that carries one or more unpaired electrons and is able to exist independently [32]. Meanwhile, free radicals have an odd number of electrons; this makes them short lived, highly reactive, and unstable. Consequently, it can react quickly with other substances trying to catch the required electron to obtain stability. Free radical can become balanced by attacking the closest stable molecule and “stealing” its electron. Meanwhile the attacked molecule can become a free radical by losing its electron and start a chain reaction cascade causing damage to the living cell [33]. Examples of free radicals are hydroxyl free radical, superoxide free radical anion, lipid peroxyl, lipid peroxide, and lipid alkoxyl. Reactive oxygen species (ROS) are radical derivatives such as singlet oxygen and hydrogen peroxide [32, 33] (Table 1).

Normal cellular metabolism produces ROS and these play crucial roles in activation of signaling pathways in animal and plant cells which alter the intra- and extracellular metabolism. Almost most of the ROS are produced in cells through the mitochondrial respiratory chain [32, 33]. During endogenous metabolic reactions, aerobic cells generate ROS (e.g., superoxide anion, hydrogen peroxide (H_2_O_2_), and hydroxyl radical and organic peroxides) as the usual products of biological diminution of molecular oxygen. Within hypoxic situation, the mitochondrial respiratory chain also generates nitric oxide (NO), which can produce other reactive nitrogen species (RNS) [32]. RNS can produce other additional reactive species, for example, reactive aldehydes-malondialdehyde and 4-hydroxynonenal, by inducing excessive lipid peroxidation. Lipids and proteins are important targets for oxidative attack and alteration of these molecules can enhance the mutagenesis process [34, 35].

In inflammatory response, leukocytes and mast cells are present in the damage regions which direct to a “respiratory burst” as a result of an enhanced uptake of oxygen and therefore enhance the production and release of ROS at the damaged area [34, 35]. However, inflammatory cells generate more soluble inflammatory mediators such as cytokines, arachidonic acid, and chemokines, which act through active inflammatory cells in the area of infection and release more reactive species. These essential markers can stimulate signal transduction cascades in addition to alterations in transcription factors, like nuclear factor kappa B (NF-*κ*B), signal transducer and activator of transcription 3, activator protein-1, NF-E2 related factor-2, nuclear factor of activated T cells, and hypoxia-inducible factor-1*α* (HIF1-*α*), which mediate vital cellular stress reactions. Initiation of cyclooxygenase-2 (COX-2), inducibility of nitric oxide synthase (iNOS), and high expression of inflammatory cytokines, including tumor necrosis factor-*α* (TNF-*α*), interleukin-1*β* (IL-1*β*), IL-6, and chemokines (CXC chemokine receptor 4), in addition to changes in the expression of specific microRNAs, have also been exhibited to have a role in oxidative stress-induced inflammation [36, 37]. This inflammatory/oxidative environment triggers an unhealthy circle, which can harm healthy stromal cells and neighboring epithelial cells, which after a long period of time may trigger carcinogenesis [33, 36].

**Figure 5:**
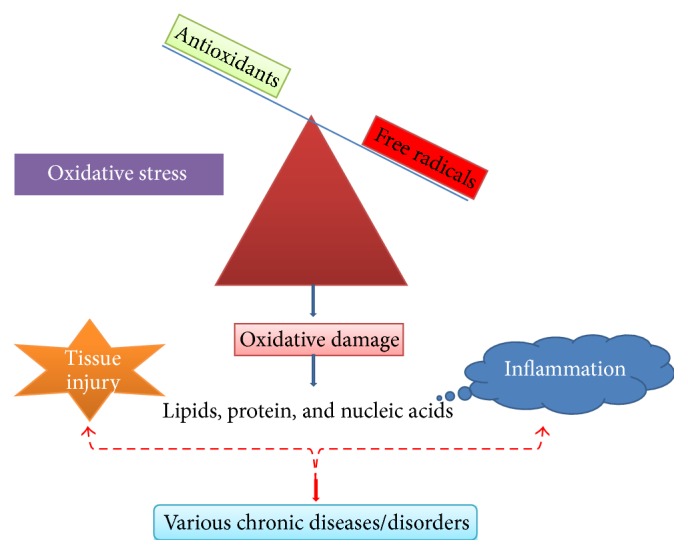
Oxidative stress and inflammation: imbalance of antioxidants and free radicals.

In normal and healthy body condition, there is a balance between reactive oxygen species formation/free radical and endogenous antioxidant defence mechanisms. However, if this equilibrium is disturbed, it can lead to oxidative stress and associated damage. This oxidative stress condition can cause injury to all vital cellular components such as DNA, proteins, and membrane lipids and it may lead to cell death [38]. As a result, it can cause numerous diseases which include diabetes, cardiovascular diseases, inflammation, cancer, degenerative diseases, ischemia, and anemia [39] (Figure 5).

### 4.2. Natural Compounds as Convenient Antioxidant and Anti-Inflammatory Agents

The World Health Organization (WHO) has estimated that 80% of the world inhabitants utilized traditional medicine for their primary health care needs and the majority of this therapy requires the use of herbal extracts and their active components. Various medicinal plant bioactive extracts and their identified/isolated active constituents have shown a variety of medicinal pharmacological properties against various acute and chronic diseases/disorders [40–49]. Currently, the impact of oxidative stress and its associated factors has become an important issue of human health [50]. When the body is under a lot of stress, the production of ROS (e.g., hydroxyl radicals, superoxide anion radicals, and hydrogen peroxide) is amplified [51]. Endogenous enzymatic and nonenzymatic antioxidant substance are not able to handle the overload of ROS and lead to imbalances of the process, cell damage [52], and health problems [53]. Lack of antioxidant compounds in daily diet can lead to the development of degenerative diseases [50] such as cancers, cardiovascular diseases [51], Alzheimer's disease, neurodegenerative diseases [54], and various inflammatory illnesses. Incorporation of antioxidant compounds by consuming natural plant sources in the daily diet can be a suitable solution to solve human health issues. These natural antioxidant sources can be used as a preventive medicine. Recent investigation suggested that there is an inverse link between the dietary consumption of antioxidant-rich foods and prevalence of human illness [50].

Numerous studies exhibited that flavonoids and phenolic content have contributed to the antioxidant activities of natural compounds. In addition, other studies also reported that trace metals such as Cu, Zn, Mg, Mn, and Se perform a significant function in antioxidant system [39]. Moreover, dietary antioxidants including tocopherols, carotenoids, and ascorbic acid have been investigated [50]. Several synthetic antioxidant supplements have been produced to remediate oxidative stress. Nevertheless, the factors such as lack of availability, high cost, and side effects remain as the main challenge in dealing with oxidative stress. For example, butylated hydroxyanisole and butylated hydroxytoluene have been widely used as a synthetic antioxidant in food industry and could possibly cause side effects like carcinogenesis and liver damage [50]. However, natural antioxidants are abundant in several plant sources, free from side effects, and less expensive. The natural compound based antioxidant substances perform a preventive role in protecting against the generation of free radicals and therefore natural based antioxidants are one of the more valuable therapeutic agents to reduce the illnesses triggered by oxidative stress [39].

Besides having antioxidant activities the flavonoids and phenolic compounds also exert an effective role as anti-inflammatory factors. The anti-inflammatory activities of natural compounds have been reported in several studies and have been observed in numerous preclinical studies [39]. The findings from anti-inflammatory researches have proven that bioactive extracts and their natural compounds exert their biological properties by blocking two major signaling pathways such as NF-*κ*B and mitogen-activated protein kinases (MAPKs) which have the main role in the production of various proinflammatory mediators. In this review article, we have discussed below the anti-inflammatory potential of medicinal plants and their mechanisms whose information was obtained from PubMed in the year between 2014 and 2015.

### 4.3. Anti-Inflammatory Potential of Medicinal Plants and Their Active Constituents

#### 4.3.1. *Acanthopanax senticosus* Harms (AS)

Dichloromethane soluble fraction showed the strongest anti-inflammatory activities through the inhibition of expression of inducible NO synthase (iNOS), COX-2, TNF-*α*, IL-1*β*, and mRNAs and the generation of reactive oxygen species in lipopolysaccharide-induced RAW 264.7 cells. AS decreased the level of NF-*κ*B and the DNA-binding activity of NF-*κ*B by inhibiting the NF-*κ*B pathway [55].

#### 4.3.2. *Actinidia arguta*

A chloroform layer of* Actinidia arguta* exerted anti-inflammatory effects via nuclear factor- (NF-) *κ*B pathway. The chloroform extract inhibits NO production and iNOS mRNA expression in RAW 264.7 cells on LPS-stimulated macrophages. Reduction in phosphorylation of mitogen-activated protein kinases including extracellular signal-regulated kinase (ERK) 1/2, c-Jun N-terminal protein kinase, and p38 was accompanied. Together, chloroform extract of* Actinidia arguta* expresses anti-inflammatory effects potential by the suppression of mitogen-activated protein kinase (MAPK) phosphorylation and nuclear translocation of NF-*κ*B in lipopolysaccharide- (LPS-) stimulated macrophages in RAW 264.7 cells [56].

#### 4.3.3. *Ainsliaea fragrans* Champ

**Figure 6:**
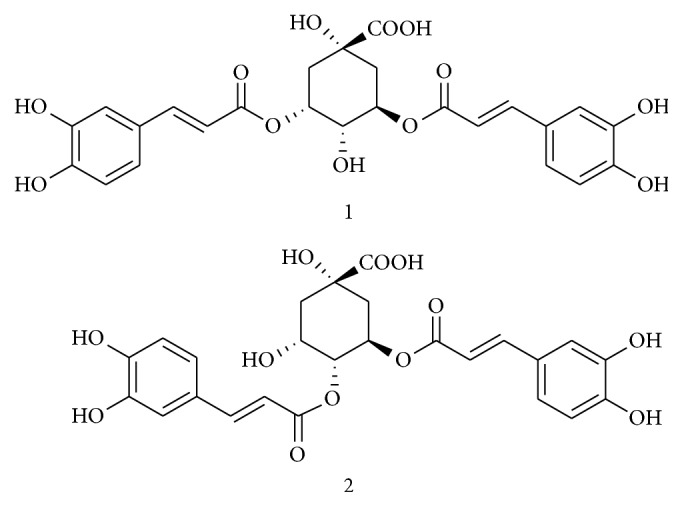
Structures of compounds 1 and 2 isolated from* A. fragrans* (3,5-dicaffeoylquinic acid and 4,5-dicaffeoylquinic acid).

*Ainsliaea fragrans* Champ. (belongs to the Asteraceae family) is a well-known herbal medicine in China and it has extensive record of medicinal practice particularly in China. It has two major active phenolic compounds including 3,5-dicaffeoylquinic acid and 4,5-dicaffeoylquinic acid (Figure 6). The extract of medicinal plant reduces the expression of key inflammatory mediators through impact on NF-*κ*B signaling pathway. The active compound, 3,5-dicaffeoylquinic acid, has effect on only inflammatory mediators but another compound 4,5-dicaffeoylquinic acid effectively inhibits the NF-*κ*B-activated pathway. In this investigation, extract and compound 4,5-dicaffeoylquinic acid have significant anti-inflammatory potential compared to another compound through suppression of signaling pathways [57].

#### 4.3.4. *Ampelopsis grossedentata*

**Figure 7:**
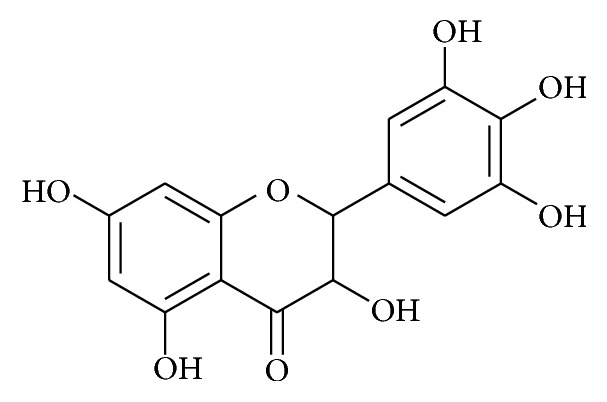
Chemical structures of dihydromyricetin.

*Ampelopsis grossedentata* (belongs to Vitaceae family) is an edible herb widely distributed in China. It is also an important traditional Chinese medicine for pharyngitis, throat infection, fever, and allergenic skin disease. Hou et al. [58] isolated the flavonoid bioactive compound, dihydromyricetin, and investigated the anti-inflammatory potential using macrophages (Figure 7). In this investigation, authors have demonstrated that this compound effectively inhibited the proinflammatory cytokines and increased the production of anti-inflammatory cytokine, IL-10. Compound, dihydromyricetin, also downregulates the inflammatory protein expression mainly iNOS and COX-2 in inflammation induced macrophage cells and this effect was achieved through suppression of the phosphorylation of NF-*κ*B and I*κ*B*α* in addition to the phosphorylation of p38 and JNK in inflammation induced macrophages. Moreover the anti-inflammatory activity of this plant is proven to hit multiple mechanisms such as ROS/Akt/I*κ*K/NF-*κ*B signaling interdependently, which is believed to be a promising therapeutic agent [59].

#### 4.3.5. *Angelica keiskei*

*Angelica keiskei* ethyl acetate-soluble fraction showed potent inhibitory action against the production of nitric oxide (NO) in LPS-activated RAW 264.7 cells. It also inhibits the LPS-induced expression of inducible nitric oxide synthase (iNOS), cyclooxygenase-2 (COX-2) genes, and PGE2 production by inhibiting the degradation of I*κ*B*α* and nuclear translocation of NF-*κ*B. The anti-inflammatory effects by HAK* Angelica keiskei* ethyl acetate-soluble fraction can be linked to interference with the signaling pathway of mitogen-activated protein kinases (MAPKs) and the activation pathway of NF-*κ*B [60].

#### 4.3.6. *Artemisiae annuae* Herba

Extract of* Artemisiae annuae* herba (AAH) inhibits NO production and various inflammatory mediators such as TNF-*α*, IL-6, and iNOS gene expression. Moreover, these extracts inhibited the nuclear translocation of p65 and I*κ*B*α* degradation in the NF-*κ*B pathway and decreased the extracellular signal-regulated kinase, p38, and c-Jun NH2-terminal kinase phosphorylation in the MAPK signaling pathway. These extracts possess the anti-inflammatory activities derived from the repression on the activation of NF-*κ*B and MAPKs pathways [61].

#### 4.3.7. *Cassia occidentalis* Roots

The bioactive compounds from* Cassia occidentalis* roots were identified and isolated from the ethyl acetate extract and they were found to suppress LPS-induced IL-1*β*, TNF-*α*, and NO production in a concentration-dependent manner in macrophages. From these active compounds, emodin and chrysophanol were also found active in inhibiting proinflammatory cytokines* in vivo* experimental model. This investigation has proven* C. occidentalis* roots extract and isolated active compounds as an effective natural therapy for the treatment and prevention of inflammation and associated ailments [62].

#### 4.3.8. *Cheilanthes albomarginata* Clarke

Various fractions were isolated from the extract and ethyl acetate fraction showed the strongest* in vitro* antioxidant properties including phenolic content, DPPH radical scavenging, hydrogen peroxide scavenging, and nitrite scavenging activity. The* in vitro* anti-inflammatory and antiadipogenic properties were measured in inflammation stimulated cells and ethyl acetate fraction showed the significant anti-inflammatory and antiadipogenic activities [63].

#### 4.3.9. *Clerodendrum inerme*

**Figure 8:**
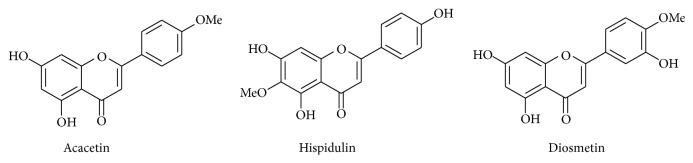
Chemical structure of acacetin (1), hispidulin (2), and diosmetin (3).

*Clerodendrum inerme* (L.) Gaertn. (belongs to Verbenaceae family) appears commonly in coastal mangrove forests of Thailand and South Asian countries as a traditional medicine. The ethyl acetate fraction of extract shows the most potent anti-inflammatory effects among other extracts/fractions through the suppression of various inflammatory markers in LPS-induced macrophages. In addition, three known flavones, acacetin (1), hispidulin (2), and diosmetin (3), were isolated based on suppression of inflammatory markers (Figure 8). Among three flavones, hispidulin is the most active anti-inflammatory agent due to suppression of NF-*κ*B DNA-binding activity and JNK signaling pathway, subsequently downregulating the key inflammatory targets such as iNOS and COX-2 expression [64].

#### 4.3.10. *Crataeva nurvala* Buch. Ham

*Crataeva nurvala* Buch. Ham. (*C. nurvala*) has been traditionally measured as medicinally important plant for treating immune function related disorders and other metabolic disorders. It has various active components which are responsible for anti-inflammatory and associated complications such as lupeol, lupeol acetate, *α*-spinasterol acetate, Ψ-taraxasterol, 3-epi-lupeol, and *β*-sitosterol as its major components and lupenone and *β*-sitosterol acetate as its minor components [65]. Suppression of various inflammatory mediators' production by the extract of* C. nurvala* was followed by downregulation of mitogen-activated protein kinases (MAPKs), specifically extracellular signal-regulated kinase (ERK) [66].

#### 4.3.11. *Cyperus rotundus*

**Figure 9:**
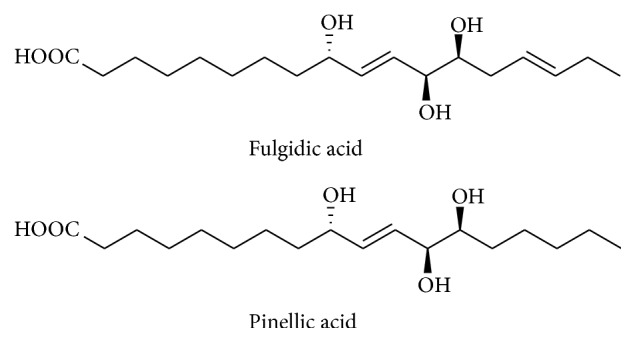
Chemical structure of fulgidic acid and pinellic acid isolated from rhizomes of* C. rotundus.*

*Cyperus rotundus* L. (Cyperaceae) is one of the traditional Chinese medicines for various diseases/disorders. Recently, researchers have isolated the bioactive compounds, namely, fulgidic acid and pinellic acid responsible for its anti-inflammatory properties (Figure 9). Fulgidic acid, an unsaturated trihydroxy C18 fatty acid, effectively suppressed the LPS-induced production of proinflammatory cytokines and various inflammatory mediators through inactivation of AP-1 transcription factor when compared to another active compound, pinellic acid [67].

#### 4.3.12. *Datura metel* L

Nine new withanolides, named daturafolisides A-I (1-9), along with six known compounds (22R) -27-hydroxy-7*α*-methoxy-1-oxowitha-3,5,24-trienolide-27-O-*β*-d-glucopyranoside, daturataturin A, daturametelin J, daturataurin B, baimantuoluoside B, and 12-deoxywithastramonolide, were identified and isolated from the leaves of* Datura metel *L. Various spectroscopic techniques including 1D and 2D NMR techniques, mass spectrometry, and circular dichroism (CD) were applied to elucidate the compounds. These isolated compounds were evaluated for* in vitro* anti-inflammatory potential using LPS-stimulated murine macrophages, and these compounds have significant anti-inflammatory properties [68].

#### 4.3.13. *Dilodendron bipinnatum* Radlk

These extracts significantly suppressed paw edema by carrageenan in the 2nd hour at 20 mg/kg and by dextran in the 1st hour at 100 mg/kg, after induction with the phlogistic agents. Besides, it has reduced total leukocytes and neutrophils migration at all different doses tested producing maximum effect at 20 mg/kg and also suppressed the concentrations of proinflammatory cytokines (IL-1*β* and TNF-*α*) and increased the level of the anti-inflammatory cytokine IL-10 in the peritonitis model [69].

#### 4.3.14. *Grateloupia lanceolata*

*Grateloupia lanceolata* is one of the seaweeds widely distributed in the seas of northeastern Asia and North America. Kim et al. [70] investigated the anti-inflammatory potential of* Grateloupia lanceolate* extract in LPS-induced RAW 264.7 cells. Extract of this plant significantly inhibited the production of proinflammatory cytokines particularly IL-1*β* expression and is related to the blockade in extracellular signal-regulated kinases 1 and 2 (ERK1/2), c-Jun N-terminal kinases 1 and 2 (JNK1/2), and NF-*κ*B signaling in LPS-stimulated RAW 264.7 cells. These findings suggest the anti-inflammatory potential of the extract in RAW 264.7 macrophages through the inhibition of LPS-stimulated p38MAPK/ERK/JNK inflammatory signaling pathways.

#### 4.3.15. *Hibiscus sabdariffa*

**Figure 10:**
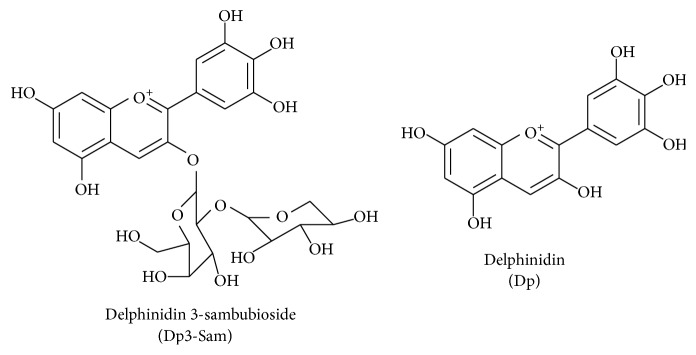
Chemical structures of delphinidin 3-sambubioside (Dp3-Sam) and delphinidin (Dp).

*Hibiscus sabdariffa* L. (Malvaceae) is a well-known functional food and Chinese herbal medicine used to treat various inflammation associated diseases. According to Sogo et al. [71] the active compound, delphinidin 3-sambubioside (Dp3-Sam), a* Hibiscus* anthocyanin from dried calices, was isolated and this compound was proven as an anticancer drug against human leukemia cells. In addition, Sogo et al. [71] discovered that anthocyanin aglycones, especially delphinidin (Dp) and cyanidin (Cy), and these compounds effectively inhibit the expression of inflammatory mediators including COX-2 and PGE2 (Figure 10). In described mechanism of active compound, delphinidin 3-sambubioside (Dp3-Sam) compound significantly downregulated the NF-*κ*B pathway and ERK1/2 signaling through suppression of numerous inflammatory mediators.

#### 4.3.16. *Houttuynia cordata*

Ethyl acetate fraction of the* H. cordata* extract downregulated the NO, PGE2, TNF-*α*, and IL-6 production in the inflammation stimulated cells and iNOS and COX-2 expression. In addition, this fraction suppressed nuclear translocation of the NF-*κ*B p65 subunit, which was associated with an inhibitory effect on I*κ*B*α* phosphorylation and also modulated the activation of MAPKs (p38 and JNK) [72].

#### 4.3.17. *Inula japonica* Thunb

**Figure 11:**
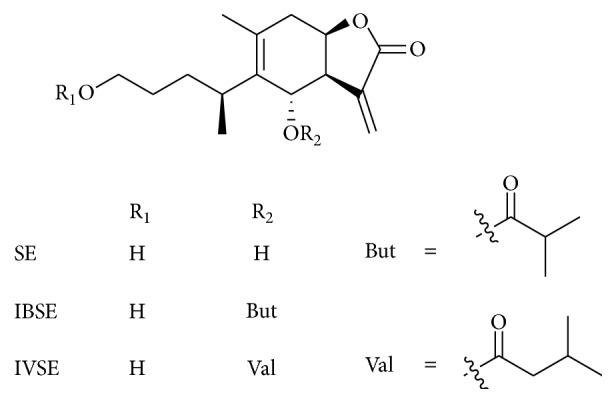
Chemical structure of SE, IBSE, and IVSE.

*Inula japonica* Thunb. (*I. japonica*) flower extract showed significant anti-inflammatory potential through preclinical studies and it has been used as one of the traditional Chinese medicines for the management of various inflammatory associated diseases. Chen et al. [73] isolated the various active compounds (1,6*α*-dihydroxy-4*α*H-1,10-secoeudesma-5(10),11(13)-dien-12,8*β*-olide (SE), 6*α*-isobutyryloxy-1-hydroxy-4*α*H-1,10-secoeudesma-5(10),11(13)-dien-12,8*β*-olide (IBSE), and 6*α*-isovaleryloxy-1-hydroxy-4*α*H-1,10-secoeudesma-5(10),11(13)-dien-12,8*β*-olide (IVSE)) from the flowers extract (Figure 11), and these compounds effectively inhibited the production of NO and PGE2 in LPS-stimulated RAW 264.7 macrophages [74].

Chen et al. [73] have indicated that active compound IVSE has a potential inhibitory effect on LPS-stimulated NO production and iNOS protein expression in macrophages and these activities might be suppression of two key signaling pathways, NF-*κ*B and MAPKs.

**Figure 12:**
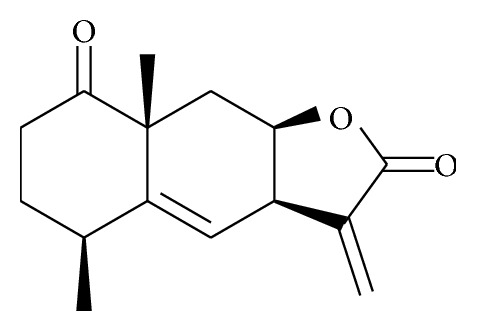
Chemical structure of JEUD-38.

Wang et al. [75] isolated the JEUD-38; 1-oxo-4aH-eudesma-5(6),11(13)-dien-12,8*β*-olide is a new sesquiterpene lactone of the plant extract (Figure 12). JEUD-38 compound also significantly inhibited NO production and proinflammatory mediators in LPS-stimulated macrophages. In addition, JEUD-38 downregulates the NF-*κ*B transcription factor through the inhibition of I*κ*B*α* phosphorylation and degradation as well as suppressing the MAPK activation.

#### 4.3.18. *Lignosus rhinocerotis*

Various extracts including cold water extract (CWE), hot water extract (HWE), and methanol extract (ME) of the sclerotial powder of* L. rhinocerotis* showed antiacute inflammatory activity by carrageenan-induced paw edema test; in this method CWE shows the most potent effect. The protein component of the high molecular weight fractions isolated from CWE contributes to the significant anti-inflammatory potential through the inhibition of TNF-*α* production with an IC_50_ of 0.76 *μ*g/mL [76].

#### 4.3.19. *Lycium barbarum* (Lycii Radicis Cortex, LRC)

The extract from plants showed anti-inflammatory potential through reduction in the LPS-induced production of inflammatory mediators such as NO and proinflammatory cytokines, IL-1*β* and IL-6, in the macrophages. Moreover, this extract inhibited the various LPS-induced inflammatory mediators such as inducible NO synthase (iNOS) and COX-2 mRNA and protein and inflammatory cytokines mRNA in the cells. The cellular mechanism of extract inspires the suppression of LPS-mediated p38 and c-Jun N-terminal kinase (JNK), mitogen-activated protein kinases (MAPKs), and the nuclear factor- (NF-) *κ*B signaling pathway in inflammation induced experimental model [77].

#### 4.3.20. *Matricaria recutita* L

*Matricaria recutita* L. belongs to Asteraceae family and has been used for centuries to treat numerous inflammatory associated diseases. Chamomile preparations were approved for oral consumption in the treatment of inflammatory diseases particularly gastrointestinal tract. The advantages of using chamomile preparations are connected to the presence of quite a few plant metabolites from various classes including flavonoids, coumarins, proazulenes, their degradation product chamazulene, and the essential oil. Among all other flavonoids, apigenin and luteolin derivatives are the most abundant and have significant* in vivo* and* in vitro* anti-inflammatory potential through modulation of various associated pathways [78].

**Figure 13:**
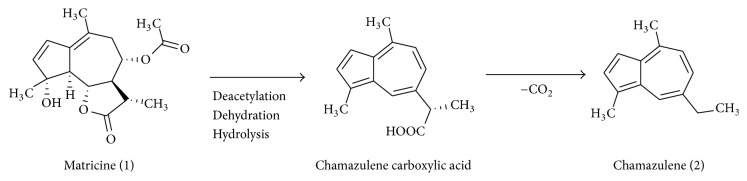
Degradation of matricine (1) to chamazulene (2) via chamazulene carboxylic acid.

The most active component is guaianolide matricine (1), with significant anti-inflammatory activities present in chamomile flower heads. Matricine (1) inhibits ICAM-1 gene, NF-*κβ* signaling molecules, and protein expression which was induced by TNF-*α* and LPS in endothelial cells. Furthermore, these inhibitions were in dose-dependent manner without causing cytotoxicity on endothelial cells. Another degradation product of matricine (1) is chamazulene (2) and this product was inactive against NF-*κ*B promotor activity [79] (Figure 13).

#### 4.3.21. *Moringa oleifera* Lam

*Moringa oleifera* Lam. (*M. oleifera*) from monogeneric family Moringaceae is well-known as “the miracle tree” and now widely distributed in many tropical and subtropical areas. All parts of the plant exhibit various important medicinal and pharmacological properties for improving human and animal health. Several researchers reported that methanolic, hydromethanolic, and hydroethanolic extracts from different parts of* M. oleifera* such as leaves, roots, pods, seeds, fruits, and flowers have possessed anti-inflammatory activities. Anti-inflammatory effects of* M. oleifera* edible parts were assessed through LPS-induced RAW 264.7 macrophages. These findings indicate that* M. oleifera* effectively suppressed the production of inflammatory mediators such as NO, proinflammatory cytokines (IL-6, IL-1*β*, and TNF-*α*), and PGE2. It has also reduced the expression of inflammatory markers which include NF-*κ*B, iNOS, and COX-2 [48, 80–83]. These findings have suggested that the edible part of* M. oleifera* may act as an effective natural anti-inflammatory remedy against inflammatory associated diseases.

#### 4.3.22. *Nuphar lutea*

*Nuphar lutea* (L.) Sm. (Nymphaeaceae family) has been broadly used for management of various inflammatory diseases. Previous investigations have proven that medicinal properties of* Nuphar* extracts have indicated numerous biomedical applications, including antileishmanial [84], antibacterial [85], and anticancer applications [86].* Nuphar lutea* extract showed significant anti-inflammatory potential through the phosphorylation of ERK1/2 and the downregulation of the NF-*κ*B signaling pathway targets, causing the alteration of cytokine production and protection of mice in two models of acute septic shock [87].

#### 4.3.23. Oryeongsan (OR)

The Oryeongsan (OR) showed anti-inflammatory potential by inhibiting various inflammatory mediators such as NO, TNF-*α*, IL-6, and IL-1*β*. Apart from this effect, it significantly suppressed COX-2, iNOS, and NO synthesizing enzymes and also induced heme oxygenase- (HO-) 1 expression and inhibited NF-*κ*B signaling pathway activation and phosphorylation of MAPKs [88].

#### 4.3.24. *Persicaria chinensis* L

*Persicaria chinensis* L. (Polygonaceae) is a medicinal herb mainly in tropical and subtropical eastern Asia and it has been used as a folk medicine in Malaysia and Nepal mainly for lung associated complication and cancer [89]. It contains various phenolic acid and flavonoids compounds such as caffeic acid, kaempferol, and quercetin which are responsible for anti-inflammatory potential. Methanolic extract of* Persicaria chinensis* effectively suppressed the production of NO and PGE2 in LPS-induced macrophages. In addition, nuclear translocation of NF-*κ*B (p65 and p50) was inhibited by extract, implying that* P*.* chinensis* extract targets Syk and Src kinases and their downstream transcription factor NF-*κ*B [90].

#### 4.3.25. *Physalis alkekengi*

*Physalis alkekengi *var.* franchetii* (PA) (Solanaceae) is a popular medicinal plant and acts as a traditional herb for the treatment of various ailments. The anti-inflammatory properties of PA were also evaluated using macrophages, RAW 264.7 cells, and OVA induced asthma in animal model. PA extract significantly downregulates the MMP9 expression due to reduced production of nitric oxide and all other proinflammatory cytokines. In addition, it has inhibited the phosphorylation of MAPKs through suppression of AP-1 signaling activation. These effects suggest that extract of PA possesses preventive and therapeutic potential for management of inflammatory associated diseases [91].

#### 4.3.26. *Porphyra yezoensis*

Edible red algae (*Porphyra yezoensis*) are commonly consumed in South East Asia and they contain few biologically functional components, dietary fibers, taurine, polyunsaturated fatty acids, carotenoids, and porphyra-334 amino acids as well as essential minerals, vitamins, and proteins. Porphyran is a major bioactive compound from this plant and it has diverse pharmacological properties including anticancer, antioxidant, and antihyperlipidemic properties. Porphyran has significant anti-inflammatory properties in a dose-dependent manner through the prevention of NF-*κ*B activation [92].

#### 4.3.27. *Saxifraga stolonifera*

**Figure 14:**
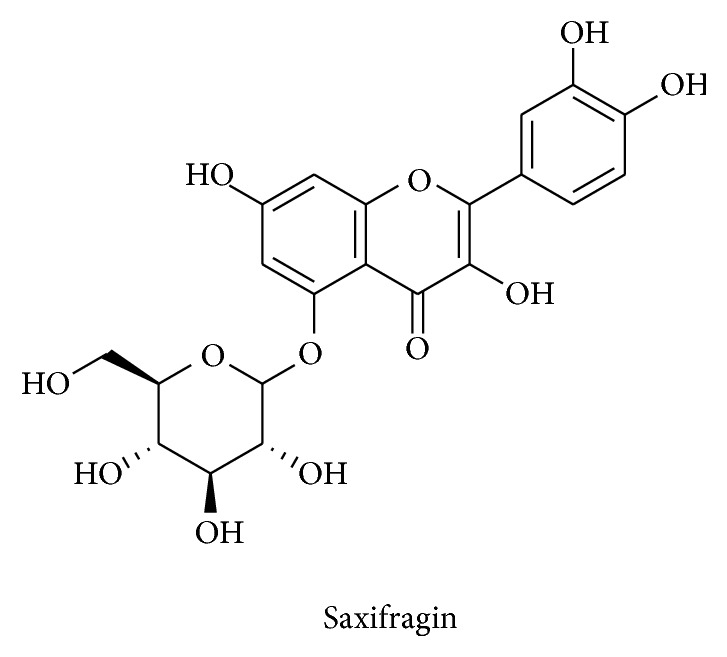
Structure of saxifragin.

*Saxifraga stolonifera* is traditional medicinal flowering plant and has higher concentration of saxifragin (quercetin-5-glucoside) (Figure 14). Saxifragin active components efficiently inhibited the LPS-stimulated nuclear translocation of p65 and activation of caspase-1 in the murine macrophages. These findings recommend that the suppressive effects of saxifragin on NF-*κ*B-regulated signaling targets in macrophages are mainly responsible for its anti-inflammatory effect in sepsis induced animal model [93]. Besides, this evergreen dicotyledon is also known for its anticancer properties and quercetin isolated is shown to induce apoptosis in human gastric cancer cell [94]. Quercetin is also an active compound to attenuate proinflammatory cytokines and mediators in macrophages [94].

#### 4.3.28. *Schisandra chinensis*

New sesquiterpenes (*β*-chamigrenic acid, *α*-ylangenol, and *α*-ylangenyl acetate) isolated from the fruit of* S. chinensis* have anti-inflammatory potential. *β*-Chamigrenal has shown most significant anti-inflammatory properties through suppression of LPS-induced NO and PGE2 in macrophages. Inhibition of nitric oxide production of *β*-chamigrenal was mediated by suppressing inducible NO synthase activity.

*β*-Chamigrenal also regulated the PGE2 synthesis pathway through upregulation of inducible microsomal prostaglandin E synthase-1 expression after stimulation with LPS. In addition, it has inhibited the expression of early growth response factor-1, a key transcription factor of microsomal prostaglandin E synthase-1 expression, implying that* S. chinensis* regulated LPS-induced NF-*κ*B-dependent inflammatory pathways via repression of MAPKs activation [95].

#### 4.3.29. *Smilax glabra* Roxb

*Smilax glabra* Roxb. (Liliaceae family) has been used as traditional medicine in both developing and developed countries. Two main fractions were eluted from the extract, namely, SGP-fr.1 and SGP-fr.2. These two fractions (SGP-1 and SGP-2) have significantly inhibited the production of various inflammatory mediators such as NO, TNF-*α*, and IL-6 from LPS-stimulated macrophages in addition to the mRNA expression of iNOS, proinflammatory cytokines, TNF-*α*, and IL-6. These inhibitions were due to the suppression of key signaling pathways, namely, extracellular signal-regulated kinase (ERK) and c-Jun NH2-terminal kinase (JNK) [96].

## 5. Summary and Conclusion

**Figure 15:**
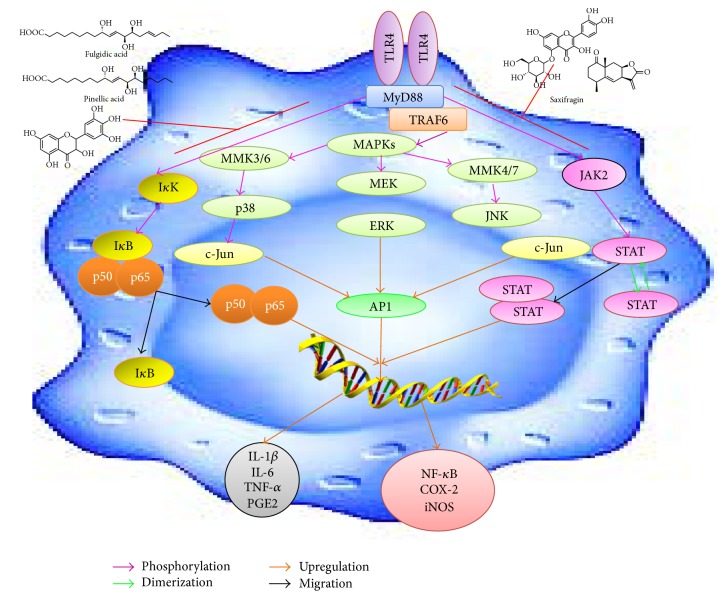
Cellular signaling inflammatory pathways and how the natural products targets inhibit the pathways.

Development of effective and economical nonsteroidal anti-inflammatory drugs (NSAIDs) with minimal or no gastrointestinal (GI) side effects is an area of importance in drug discovery pharmaceutical industry. The use of the most frequently recommended drugs of analgesics such as aspirin currently is restricted because of their possible side effects such as severe gastric disorders and other GI side effects. Anti-inflammatory drugs include “biologicals” like anticytokine therapies which block the activity of various kinases and show a significant decrease in host defence toward infections. Due to these side effects and health problems of existing anti-inflammatory therapies, natural anti-inflammatory supplements are becoming more popular and many scientific investigations have been concentrated on this area. In recent years, enormous deal of effort has focused on using available experimental techniques to verify natural antioxidant and anti-inflammatory drugs from natural product resources. This review article will provide scientific knowledge toward anti-inflammatory drug development for various chronic inflammatory associated diseases/disorders from natural products (Figure 15).

## Figures and Tables

**Table 1:** List of free radicals and their reactivity.

No.	Free radicals	Reactivity
1	Superoxide anion	Generated in mitochondria, cardiovascular system, and other cell types
2	Hydrogen peroxide	Formed in the human body by a large number of reactions and yields potent reactive species
3	Hydroxyl radical	Highly reactive and generated during iron overload and such conditions in the human body
4	Peroxyl radical	Reactive and formed from lipids, proteins, DNA, and sugar molecules during oxidative damage
5	Nitric oxide	Neurotransmitter and blood pressure regulation and can yield potent oxidants during pathological states
6	Peroxynitrite	Highly reactive and formed from NO and superoxide
7	Ozone	Present as an atmospheric pollutant and can react with various molecules

## Abbreviations

IL: Interleukin
TNF-*α*: Tumor necrosis factor-alpha
PGE2: Prostaglandin E2
NF-*κ*B: Nuclear factor kappa B
COX-2: Cyclooxygenase-2
iNOS: Inducible nitric oxide synthase
TLR: Toll-like receptor
MyD: Myeloid differentiation primary response gene
STAT: Signal transducer and activator of transcription
AP: Activation protein
I*κ*B: Inhibitor of nuclear factor kappa B
I*κ*K: Inhibitor of nuclear factor kappa B kinase
TRAF: TNF receptor associated factor
MAPKs: Mitogen-activated protein kinases
MEK/MKK/p38: Mitogen-activated protein kinase
ERK: Extracellular signal-regulated kinase
JAK: Janus kinase
JNK: c-Jun N-terminal kinase.
